# Familial hypercholesterolaemia in UK primary care: a Clinical Practice Research Datalink study of an under-recognised condition

**DOI:** 10.3399/BJGP.2023.0010

**Published:** 2024-02-06

**Authors:** Adeline Durand, Christopher Ll Morgan, Steven Tinsley, Elizabeth Hughes, Terry McCormack, Charlotte L Bitchell, Raquel Lahoz

**Affiliations:** Novartis UK Ltd, London, UK.; Pharmatelligence, Cardiff, UK.; Novartis UK Ltd, London, UK.; Sandwell and West Birmingham Hospitals NHS Trust and University of Aston Medical School, Birmingham, UK.; Institute of Clinical and Applied Health Research, Hull York Medical School, Hull, UK.; Pharmatelligence, Cardiff, UK.; Novartis AG, Basel, Switzerland.

**Keywords:** familial hypercholesterolaemia, hypercholesterolaemia, lipid-lowering therapy, prevalence, primary health care

## Abstract

**Background:**

Studies utilising genotyping methods report the prevalence of familial hypercholesterolaemia to be as high as one in 137 of the adult population.

**Aim:**

To estimate the prevalence of familial hypercholesterolaemia measured by clinically coded diagnosis, associated treatments, and lipid measurements observed in UK primary care.

**Design and setting:**

This was a retrospective analysis using the Clinical Practice Research Datalink (CPRD) GOLD database.

**Method:**

Patients aged ≥18 years and actively registered on the index date (30 June 2018) formed the study cohort. Point prevalence of familial hypercholesterolaemia for 2018 was estimated overall and for each nation of the UK. Patients with familial hypercholesterolaemia were stratified into primary and secondary prevention groups, defined as those with/without a prior diagnosis of atherosclerotic cardiovascular disease. Prevalence estimates and extrapolations were replicated for these subgroups. Baseline demographic, lipid, and clinical characteristics for the prevalent cohort were presented.

**Results:**

In total, 4048 patients with familial hypercholesterolaemia formed the study cohort. The estimated familial hypercholesterolaemia prevalence for the UK was 16.4 per 10 000 (95% confidence interval [CI] = 16.0 to 16.9). Of these, 2646 (65.4%) patients with familial hypercholesterolaemia had a recent prescription for lipid-lowering therapy. Mean lipid levels were lower for those treated with lipid-lowering therapy compared with those untreated: 5.34 mmol/L (SD 1.50) versus 6.25 mmol/L (SD 1.55) for total cholesterol and 3.15 mmol/L (SD 1.34) versus 3.96 mmol/L (SD 1.36) for low-level density lipoprotein cholesterol.

**Conclusion:**

The estimated prevalence of familial hypercholesterolaemia was one in 608 of the population, less than expected from other studies, which may indicate that familial hypercholesterolaemia is under-recognised in UK primary care. Over one-third of diagnosed patients were undertreated and many did not achieve target goals, placing them at risk of cardiovascular events.

## Introduction

Familial hypercholesterolaemia is a mono-and polygenic condition manifested by high cholesterol, particularly elevated low-level density lipoprotein cholesterol (LDL-C).^1^ The National Institute for Health and Care Excellence (NICE) guidelines^2^ suggest that familial hypercholesterolaemia should be suspected when a patient presents with total cholesterol >7.5 mmol/L and/or where a coronary heart disease (CHD) event has been reported in a first-degree relative before the age of 60 years. Physicians are assisted in the diagnosis of familial hypercholesterolaemia by algorithms such as the Simon Broome Register^3^ or the Dutch Lipid Clinic Network criteria,^4^ which attribute the probability of a familial hypercholesterolaemia diagnosis on to the presence of genetic mutation, elevated LDL-C, tendon xanthoma, and a family history of either hypercholesterolaemia or premature myocardial infarction.

Historically, the prevalence of familial hypercholesterolaemia has been reported as approximately one in 500 of the population^2^^,^^5^ but a recent meta-analysis has estimated that the prevalence of heterozygous familial hypercholesterolaemia is considerably higher at one in 311.^6^ Homozygous familial hypercholesterolaemia is estimated to occur in one in 1 million of the population.^7^^,^^8^ Estimating the prevalence of familial hypercholesterolaemia in the general population is difficult because of the necessity for genetic screening; however, studies utilising genotyping methods have reported the prevalence of familial hypercholesterolaemia as high as one in 137 of the adult population.^9^ Regardless of the true population prevalence, it is likely that, across different contexts, familial hypercholesterolaemia is underdiagnosed.^10^ This is partly because patients with familial hypercholesterolaemia are initially asymptomatic and, in younger age groups, may not be as likely to be screened asymptomatically. Within the primary care setting, access to appropriate genetic screening case-identification methods such as cascade screening requires significant resource.

**Table:** How this fits in

Studies utilising genotyping methods have reported the prevalence of familial hypercholesterolaemia to be as high as one in 137 of the population. Familial hypercholesterolaemia is associated with high risk of experiencing cardiovascular events at a relatively young age. Early identification of patients allows for earlier intervention. The current study estimated the prevalence of familial hypercholesterolaemia as one patient per 608 population, suggesting significant underdiagnosis in primary care. Over one-third of patients had no evidence of lipid-lowering therapies prescribed within 6 months of the study index date and this was associated with elevated levels of low-density lipoprotein cholesterol. Failure to identify patients with the condition and suboptimal treatments represent a lost opportunity to delay or prevent cardiovascular events in those with familial hypercholesterolaemia.

As familial hypercholesterolaemia is present from birth and long-term, cumulative exposure to elevated LDL-C is associated with the development of atherosclerosis, patients with familial hypercholesterolaemia have an extremely high risk of experiencing cardiovascular events at a relatively young age.^11^^,^^12^ It is imperative to identify and treat patients with familial hypercholesterolaemia as early as possible; however, there is evidence that many patients are diagnosed late, reducing opportunities for early interventions.^13^ The portal for case identification is primary care, with GPs required to screen patients with total cholesterol >7.5 mmol/L (aged <30 years) or >9.5 mmol/L where the patient is aged ≥30 years.^3^

There is evidence that lipid-lowering therapies such as statins may reduce cardiovascular events in individuals with familial hypercholesterolaemia.^11^^,^^14^ However, patients with familial hypercholesterolaemia are particularly difficult to treat with reportedly only 44% of UK adult patients achieving NICE guideline goals of a 50% reduction in LDL-C from untreated levels.^15^

This study aimed to determine the prevalence of recognised familial hypercholesterolaemia as recorded within primary care electronic health records using clinical codes and to estimate how many of those patients were receiving lipid-lowering therapy.

## Method

### Data sources

This was a retrospective analysis of patients with familial hypercholesterolaemia within the Clinical Practice Research Datalink (CPRD) GOLD,^16^ a longitudinal research database derived from primary care practices using the VISION^17^ software for practice administration. The database contains data on demographics, diagnoses, prescriptions, and other aspects of patient care. Diagnostic information in CPRD GOLD is recorded using the Read classification.^18^

### Patient selection

Point prevalence of familial hypercholesterolaemia was estimated for 2018 to align with the requirements of a related study. Patients with data of acceptable research quality, actively registered, and aged ≥18 years on the index date of 30 June 2018 formed the denominator population. Patients with a first diagnosis of familial hypercholesterolaemia recorded using the Read code classification (C320000, C320.11) in the CPRD GOLD clinical or referral tables before 30 June 2018 (inclusive) formed the numerator. The prevalence rate with 95% confidence intervals (CIs) was provided for the population overall and stratified for each constituent nation of the UK. To estimate the number of people with familial hypercholesterolaemia in each nation, observed 2018 prevalence rates were applied to the Office for National Statistics population estimates for the UK 2018.^19^ Owing to the imbalance in the representation of the different nations of the UK within CPRD GOLD, weighted rates adjusting for the different population sizes were applied to estimate the prevalence of familial hypercholesterolaemia in the UK. Patients with familial hypercholesterolaemia were stratified into primary prevention and secondary prevention, defined as those with or without a prior diagnosis of CHD, cerebrovascular disease or peripheral arterial disease (PAD), respectively. All prevalence estimates and extrapolations were replicated in these subgroups.

Baseline demographic, lipid, and clinical characteristics for the prevalent cohort were presented. Ten-year cardiovascular risk was assessed using the Framingham,^20^ SCORE2,^21^ and American College of Cardiology (ACC)/American Heart Association (AHA)^22^ algorithms. The nearest contributing variables to 30 June 2018 were used to calculate the risk scores, with modifiable variables (cholesterol and blood pressure markers) limited to those reported in the preceding 3 years. Smoking history was based on the last recorded value in the database. Patients with a prescription of a lipid-lowering therapy (statins, ezetimibe, fibrates, proprotein convertase subtilisin/kexin type 9 [PCSK9] inhibitors, niacin, and bile acid sequestrants) identified from the CPRD GOLD therapy table in the 6 months before 30 June 2018 were flagged. Summary data detailed prescription of statins (including by statin intensity,^23^ monotherapy, and combination with ezetimibe), ezetimibe, and fibrates, in addition to overall lipid-lowering therapy use. Those patients prescribed statins before, but not during, the 6-month window were flagged as potentially statin intolerant/contraindicated.

## Results

**Figure 1.**
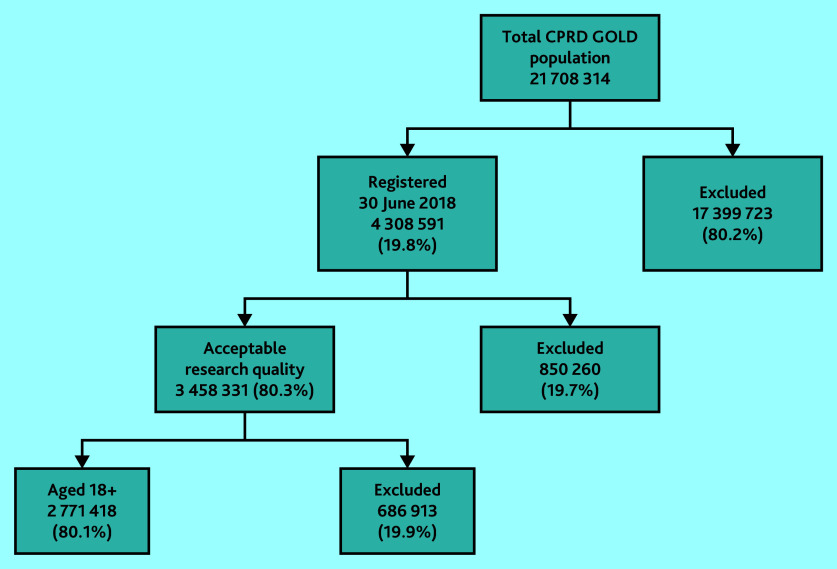
Derivation of Clinical Practice Research Datalink (CPRD) GOLD denominator population (30 June 2018) for the estimation of prevalence of familial hypercholesterolaemia.

Within the July 2020 build of CPRD GOLD, there were 21 708 314 patients. Of these 4 308 591 (19.8%) had a current registration with a participating primary care practice on 30 June 2018. After application of the study inclusion and exclusion criteria, 2 771 418 patients formed the denominator population (Figure 1).

There were 4048 patients who had a recorded diagnosis of familial hypercholesterolaemia before or on 30 June 2018. This equated to a prevalence within the CPRD GOLD database of 14.6 (95% CI = 14.2 to 15.1) per 10 000 population, ranging from 11.4 (95% CI = 10.8 to 12.1) in Scotland to 17.1 (95% CI = 16.2 to 17.9) in England (Table 1). After adjusting for the imbalance of the different nations, the estimated prevalence for the UK was 16.4 per 10 000 (95% CI = 16.0 to 16.9). Of those patients with familial hypercholesterolaemia, the majority of patients (85.2%) were classified as primary prevention group, ranging from 81.4% in Scotland to 88.4% in England.

**Table 1.** Estimated prevalence of familial hypercholesterolaemia^a^ and extrapolated patients for England, Northern Ireland, Scotland, and Wales, and weighted prevalence for the UK in the CPRD GOLD database 2018

**Nation**	**All patients**			**Primary prevention FH**			**Secondary prevention FH**		
	**Patients with FH, *n***	**Prevalence per 10 000 (95% CI)**	**Extrapolated cases of FH, *n***	**Primary prevention FH cases**	**Prevalence per 10 000 (95% CI)**	**Primary prevention FH extrapolated**	**Secondary prevention FH cases**	**Prevalence per 10 000 (95% CI)**	**Secondary prevention FH extrapolated**
**England**	1512	17.1 (16.2 to 17.9)	75 095	1336 (88.4)	15.1 (14.3 to 15.9)	66 354	176 (11.6)	2.0 (1.7 to 2.3)	8741

**Northern Ireland**	277	13.4 (11.8 to 15.0)	1935	238 (85.9)	11.5 (10.1 to 13.0)	1663	39 (14.1)	1.9 (1.3 to 2.5)	272
**Scotland**	1126	11.4 (10.8 to 12.1)	5041	953 (84.6)	9.7 (9.1 to 10.3)	4267	173 (15.4)	1.8 (1.5 to 2.0)	775
**Wales**	1133	16.3 (15.4 to 17.3)	4098	922 (81.4)	13.3 (12.4 to 14.1)	3335	211 (18.6)	3.0 (2.6 to 3.5)	763
**Total CPRD**	4048	14.6 (14.2 to 15.1)	76 513	3449 (85.2)	12.4 (12.0 to 12.9)	65 191	599 (14.8)	2.2 (2.0 to 2.3)	11 322
**Weighted UK^b^**	4559	16.4 (16.0 to 16.9)	86 170	4001 (87.8)	14.4 (14.0 to 14.9)	75 618	558 (12.2)	2.0 (1.8 to 2.2)	10 551

a *Prevalent cases defined as patients with a recorded diagnosis code for familial hypercholesterolaemia within the CPRD GOLD database.*

b *Estimate prevalence adjusted to the proportion of patients by nation within the CPRD GOLD database. CI = confidence interval. CPRD = Clinical Practice Research Datalink.* *FH = familial hypercholesterolaemia.*

Baseline characteristics for the familial hypercholesterolaemia population overall and by nation are shown in Table 2. Overall mean age was 57.7 (SD 14.6) years, and 2326 (57.5%) patients were female. There was a history of myocardial infarction for 4.7% of patients, ischaemic stroke for 3.4%, and 1.7% for PAD. Of these patients, 13.3% were current smokers. Median 10-year cardiovascular risk ranged from 4.2% using the SCORE2 algorithm to 12.8% using the Framingham algorithm.

**Table 2.** Baseline characteristics of prevalent familial hypercholesterolaemia population^a^ for England, Northern Ireland, Scotland, and Wales, and the UK in the CPRD GOLD database 2018

**Characteristic**	**England**	**Northern Ireland**	**Scotland**	**Wales**	**UK**
**Patients, *n***	1512	277	1126	1133	4048
Males	661 (43.7)	120 (43.3)	484 (43.0)	457 (40.3)	1722 (42.5)
Females	851 (56.3)	157 (56.7)	642 (57.0)	676 (59.7)	2326 (57.5)

**Age, years, mean (SD)**	57.6 (13.6)	52.6 (16.5)	58.5 (14.7)	58.3 (15.1)	57.7 (14.6)

**Systolic blood pressure**					
Not treated with antihypertensive					
*n* (%)	682 (45.1)	117 (42.2)	438 (38.9)	451 (39.8)	1688 (41.7)
Mean (SD)	128.3 (14.2)	126.4 (13.8)	128.2 (14.5)	129.3 (14.7)	128.4 (14.4)

Treated with antihypertensive					
*n* (%)	541 (35.8)	113 (40.8)	459 (40.8)	496 (43.8)	1609 (39.7)
Mean (SD)	132.4 (14.7)	133.9 (17.1)	133.6 (15.0)	133.7 (15.6)	133.2 (15.2)

**Baseline morbidity, *n* (%)**					
Type 2 diabetes	117 (7.7)	20 (7.2)	81 (7.2)	118 (10.4)	336 (8.3)
Myocardial infarction	46 (3.0)	10 (3.6)	63 (5.6)	72 (6.4)	191 (4.7)
Ischaemic stroke	38 (2.5)	9 (3.2)	47 (4.2)	42 (3.7)	136 (3.4)
Peripheral arterial disease	12 (0.8)	10 (3.6)	25 (2.2)	22 (1.9)	69 (1.7)

**Smoking history, *n* (%)**					
Non-smoker	860 (56.9)	165 (59.6)	629 (55.9)	636 (56.1)	2290 (56.6)
Ex-smoker	457 (30.2)	74 (26.7)	316 (28.1)	332 (29.3)	1179 (29.1)
Current smoker	186 (12.3)	38 (13.7)	166 (14.7)	150 (13.2)	540 (13.3)
Missing	9 (0.6)	0 (0.0)	15 (1.3)	15 (1.3)	39 (1.0)

**Cardiovascular risk scores**					

Framingham					
*n* (%)	1003 (66.3)	180 (65.0)	724 (64.3)	821 (72.5)	2728 (67.4)
Mean (SD)	15.1 (12.5)	14.3 (12.0)	16.6 (12.9)	16.8 (13.3)	16.0 (12.8)
Median (IQR)	11.8 (6.7–19.7)	10.8 (6.0–18.9)	13.4 (7.6 to 21.3)	13.8 (7.8–22.5)	12.8 (7.1–20.8)

SCORE2					
*n* (%)	1003 (66.3)	180 (65.0)	724 (64.3)	821 (72.5)	2728 (67.4)
Mean (SD)	5.8 (6.3)	5.5 (6.4)	6.6 (6.9)	6.8 (7.0)	6.3 (6.7)
Median (IQR)	3.9 (1.6–7.4)	3.8 (1.3–7.3)	4.3 (2.1–9.1)	4.8 (2.0–9.4)	4.2 (1.8–8.5)

ACC/AHA					
*n* (%)	1003 (66.3)	180 (65.0)	724 (64.3)	821 (72.5)	2728 (67.4)
Mean (SD)	12.4 (14.3)	12.3 (14.9)	14.6 (15.9)	15.2 (16.9)	13.8 (15.6)
Median (IQR)	7.8 (2.9–16.1)	8.1 (2.4–16.7)	9.2 (4.2–19.8)	9.8 (3.9–20.5)	8.6 (3.4–18.3)

a *Prevalent cases defined as patients with a recorded diagnosis code for familial hypercholesterolaemia within the CPRD GOLD database. ACC/AHA = American College of Cardiology/American Heart Association. CPRD = Clinical Practice Research Datalink GOLD. IQR = interquartile range. SD = standard deviation.*

There were 2646 (65.4%) patients who had a record of a prescription for lipid-lowering therapy in the 6 months before the index date. This was lower for those classified in the primary prevention group (2110, 61.2%) versus those in the 89.5% (Table 3).

**Table 3.** Number of patients with familial hypercholesterolaemia^a^ prescribed lipid-lowering therapies by nation and overall, in the CPRD GOLD database 2018

**Therapy**	**England, *n* (%)**	**Northern Ireland, *n* (%)**	**Scotland, *n* (%)**	**Wales, *n* (%)**	**UK, *n* (%)**
**All patients**	1512	277	1126	1133	4048
Statin	896 (59.3)	167 (60.3)	702 (62.3)	776 (68.5)	2541 (62.8)
Statin monotherapy	741 (49.0)	107 (38.6)	592 (52.6)	578 (51.0)	2018 (49.9)
Low-intensity statin	22 (1.5)	<5	21 (1.9)	26 (2.3)	—
Medium-intensity statin	530 (35.1)	68 (24.5)	397 (35.3)	403 (35.6)	1398 (34.5)
High-intensity statin	344 (22.8)	98 (35.4)	284 (25.2)	347 (30.6)	1073 (26.5)
Statin intolerant	251 (16.6)	46 (16.6)	187 (16.6)	179 (15.8)	663 (16.4)
Ezetimibe	128 (8.5)	57 (20.6)	91 (8.1)	176 (15.5)	452 (11.2)
Ezetimibe monotherapy	12 (0.8)	5 (1.8)	5 (0.4)	8 (0.7)	30 (0.7)
Statin intolerant and ezetimibe	11 (0.7)	5 (1.8)	7 (0.6)	13 (1.1)	36 (0.9)
Statin + ezetimibe	113 (7.5)	52 (18.8)	82 (7.3)	161 (14.2)	408 (10.1)
Fibrates	27 (1.8)	9 (3.2)	25 (2.2)	39 (3.4)	100 (2.5)
Any lipid-lowering therapy	925 (61.2)	176 (63.5)	730 (64.8)	815 (71.9)	2646 (65.4)

**Primary prevention**	1336	238	953	922	3449
Statin	743 (55.6)	131 (55.0)	557 (58.4)	597 (64.8)	2028 (58.8)
Statin monotherapy	623 (46.6)	84 (35.3)	475 (49.8)	456 (49.5)	1638 (47.5)
Low-intensity statin	21 (1.6)	<5	17 (1.8)	21 (2.3)	—
Medium-intensity statin	464 (34.7)	57 (23.9)	329 (34.5)	335 (36.3)	1185 (34.4)
High-intensity statin	258 (19.3)	73 (30.7)	211 (22.1)	241 (26.1)	783 (22.7)
Statin intolerant	233 (17.4)	44 (18.5)	163 (17.1)	152 (16.5)	592 (17.2)
Ezetimibe	102 (7.6)	46 (19.3)	69 (7.2)	126 (13.7)	343 (9.9)
Ezetimibe monotherapy	11 (0.8)	5 (2.1)	5 (0.5)	5 (0.5)	26 (0.8)
Statin intolerant and ezetimibe	10 (0.7)	5 (2.1)	6 (0.6)	7 (0.8)	28 (0.8)
Statin + ezetimibe	89 (6.7)	41 (17.2)	61 (6.4)	118 (12.8)	309 (9.0)
Fibrates	18 (1.3)	5 (2.1)	17 (1.8)	25 (2.7)	65 (1.9)
Any lipid-lowering therapy	768 (57.5)	140 (58.8)	579 (60.8)	623 (67.6)	2110 (61.2)

**Secondary prevention**	176	39	173	211	599
Statin	153 (86.9)	36 (92.3)	145 (83.8)	179 (84.8)	513 (85.6)
Statin monotherapy	118 (67.0)	23 (59.0)	117 (67.6)	122 (57.8)	380 (63.4)
Low-intensity statin	<5	0 (0.0)	<5	5 (2.4)	—
Medium-intensity statin	66 (37.5)	11 (28.2)	68 (39.3)	68 (32.2)	213 (35.6)
High-intensity statin	86 (48.9)	25 (64.1)	73 (42.2)	106 (50.2)	290 (48.4)
Statin intolerant	18 (10.2)	2 (5.1)	24 (13.9)	27 (12.8)	71 (11.9)
Ezetimibe	26 (14.8)	11 (28.2)	22 (12.7)	50 (23.7)	109 (18.2)
Ezetimibe monotherapy	<5	0 (0)	0 (0.0)	<5	—
Statin intolerant and ezetimibe	<5	0 (0)	<5	6 (2.8)	—
Statin + ezetimibe	24 (13.6)	11 (28.2)	21 (12.1)	43 (20.4)	99 (16.5)
Fibrates	9 (5.1)	4 (10.3)	8 (4.6)	14 (6.6)	35 (5.8)
Any lipid-lowering therapy	157 (89.2)	36 (92.3)	151 (87.3)	192 (91.0)	536 (89.5)

a *Prevalent cases defined as patients with a recorded diagnosis code for familial hypercholesterolaemia within the CPRD GOLD database. In accordance with CPRD guidelines all cells with*n*<5 are suppressed. CPRD = Clinical Practice Research Datalink.*

Wales (71.9%) had the highest proportion of patients with a record of lipid-lowering therapy prescribed whereas England had the lowest (61.2%). Statins were the most commonly prescribed therapy. A record for a prescription of a statin was present for 2541 (62.8%) patients: 2028 (58.8%) of those classified in the primary prevention group and 513 (85.6%) of those classified in the secondary prevention group. Based on the definition used in the current study, 663 (16.4%) patients were classified as being statin intolerant/contraindicated; this was higher in those classified in the primary prevention group (592, 17.2%) when compared with patients classified in the secondary prevention group (71, 11.9%). Ezetimibe was prescribed overall for 452 (11.2%x) patients, but the proportion of patients prescribed ezetimibe ranged from 91 (8.1%) in Scotland, 128 (8.5%) in England, 176 (15.5%) in Wales, to 20.6% in Northern Ireland.

Baseline lipids are shown in Table 4. Overall, the majority of patients had at least one lipid measurement recorded: 3252 (80.6%) had a recording of total cholesterol, 2628 (64.9%) had LDL-C, and 3142 (77.6%) had high-level density lipoprotein cholesterol (HDL-C). Although the proportion of patients with a total cholesterol measurement recorded was similar between nations, ranging from 865 (76.8%) in Scotland to 940 (83.0%) in Wales, there was far wider variation for LDL-C, ranging from 537 (47.7%) in Scotland to 940 (80.1%) in Wales. Overall mean total cholesterol levels were 5.60 mmol/L (SD 1.57), LDL-C was 3.37 mmol/L (SD 1.39), HDL was 1.44 mmol/L (SD 0.44), and triglycerides were 2.05 (SD 1.50).

**Table 4.** Total cholesterol, low-density lipoprotein, and high-density lipoprotein for patients with familial hypercholesterolaemia^a^ overall and by treatments status in the CPRD GOLD database 2018

**Treatment**	**England**	**Northern Ireland**	**Scotland**	**Wales**	**UK**
**All patients**	1512	277	1126	1133	4048

Total cholesterol					
*n* (%)	1244 (82.3)	213 (76.9)	865 (76.8)	940 (83.0)	3262 (80.6)
mmol/L, mean (SD)	5.70 (1.58)	5.43 (1.64)	5.62 (1.58)	5.47 (1.52)	5.60 (1.57)
mmol/L, median (IQR)	5.50 (4.60–6.50)	5.10 (4.30–6.40)	5.39 (4.60–6.45)	5.20 (4.50–6.20)	5.30 (4.57–6.40)

Low-density lipoprotein					
*n* (%)	981 (64.9)	203 (73.3)	537 (47.7)	907 (80.1)	2628 (64.9)
mmol/L, mean (SD)	3.45 (1.43)	3.27 (1.57)	3.42 (1.42)	3.27 (1.28)	3.37 (1.39)
mmol/L, median (IQR)	3.20 (2.41–4.20)	2.80 (2.20–3.90)	3.20 (2.40–4.20)	3.00 (2.40–3.90)	3.10 (2.40–4.10)

High-density lipoprotein					
*n* (%)	1178 (77.9)	198 (71.5)	836 (74.2)	930 (82.1)	3142 (77.6)
mmol/L, mean (SD)	1.47 (0.46)	1.45 (0.41)	1.43 (0.45)	1.40 (0.41)	1.44 (0.44)
mmol/L, median (IQR)	1.40 (1.13–1.73)	1.40 (1.16–1.70)	1.33 (1.10–1.68)	1.30 (1.10–1.60)	1.38 (1.10–1.70)

**Triglycerides**					
*n* (%)	1059 (70.0)	197 (71.1)	594 (52.8)	915 (80.8)	2765 (68.3)
mmol/L, mean (SD)	2.06 (1.55)	1.93 (1.52)	2.14 (1.62)	2.01 (1.35)	2.05 (1.50)
mmol/L, median (IQR)	1.60 (1.07–2.44)	1.45 (1.03–2.32)	1.67 (1.16–2.50)	1.70 (1.10–2.40)	1.60 (1.10–2.43)

**All patients treated with LLT^b^**	925	176	730	815	2646

Total cholesterol					
*n* (%)	848 (91.7)	147 (83.5)	615 (84.2)	733 (89.9)	2343 (88.5)
mmol/L, mean (SD)	5.37 (1.45)	5.24 (1.66)	5.41 (1.54)	5.27 (1.48)	5.34 (1.50)
mmol/L, median (IQR)	5.10 (4.40–6.00)	4.84 (4.20–5.90)	5.10 (4.50–6.10)	5.00 (4.40–5.80)	5.10 (4.40–5.90)

Low-density lipoprotein					
*n* (%)	671 (72.5)	139 (79.0)	400 (54.8)	708 (86.9)	1918 (72.5)
mmol/L, mean (SD)	3.16 (1.35)	3.09 (1.57)	3.29 (1.45)	3.07 (1.19)	3.15 (1.34)
mmol/L, median (IQR)	2.90 (2.22–3.64)	2.66 (2.10–3.60)	3.00 (2.30–3.90)	2.80 (2.20–3.60)	2.90 (2.28–3.70)

High-density lipoprotein					
*n* (%)	802 (86.7)	135 (76.7)	595 (81.5)	728 (89.3)	2260 (85.4)
mmol/L, mean (SD)	1.47 (0.45)	1.43 (0.40)	1.40 (0.45)	1.40 (0.42)	1.43 (0.44)
mmol/L, median (IQR)	1.40 (1.16–1.72)	1.39 (1.13–1.67)	1.30 (1.10–1.64)	1.30 (1.10–1.60)	1.36 (1.10–1.69)

Triglycerides					
*n* (%)	736 (79.6)	134 (76.1)	443 (60.7)	716 (87.9)	2029 (76.7)
mmol/L, mean (SD)	2.13 (1.58)	2.01 (1.60)	2.22 (1.72)	2.07 (1.43)	2.12 (1.56)
mmol/L, median (IQR)	1.70 (1.10–2.50)	1.51 (1.0–2.48)	1.70 (1.17–2.59)	1.70 (1.10–2.50)	1.7 (1.10–2.50)

**All patients not treated with LLT^b^**	587	101	396	318	1402

Total cholesterol					
*n* (%)	396 (67.5)	66 (65.3)	250 (63.1)	207 (65.1)	919 (65.5)
mmol/L, mean (SD)	6.41 (1.61)	5.87 (1.51)	6.16 (1.55)	6.19 (1.42)	6.25 (1.55)
mmol/L, median (IQR)	6.35 (5.50–7.20)	5.66 (4.50–6.80)	6.20 (4.93–7.00)	6.00 (5.20–7.00)	6.20 (5.20–7.10)

Low-density lipoprotein					
*n* (%)	310 (52.8)	64 (63.4)	137 (34.6)	199 (62.6)	710 (50.6)
mmol/L, mean (SD)	4.08 (1.39)	3.66 (1.50)	3.80 (1.27)	3.99 (1.30)	3.96 (1.36)
mmol/L, median (IQR)	3.98 (3.20–4.80)	3.62 (2.51–4.60)	3.80 (2.80–4.62)	3.80 (3.20–4.80)	3.80 (3.10–4.70)

High-density lipoprotein					
*n* (%)	376 (64.1)	63 (62.4)	241 (60.9)	202 (63.5)	882 (62.9)
mmol/L, mean (SD)	1.47 (0.47)	1.50 (0.44)	1.48 (0.44)	1.38 (0.39)	1.45 (0.45)
mmol/L, median (IQR)	1.40 (1.10–1.75)	1.40 (1.15–1.83)	1.38 (1.15–1.72)	1.30 (1.10–1.60)	1.39 (1.10–1.70)

Triglycerides					
*n* (%)	323 (55.0)	63 (62.4)	151 (38.1)	199 (62.6)	736 (52.5)
mmol/L, mean (SD)	1.92 (1.48)	1.77 (1.35)	1.92 (1.27)	1.81 (0.97)	1.88 (1.30)
mmol/L, median (IQR)	1.50 (1.00–2.30)	1.32 (0.98–2.10)	1.56 (1.15–2.40)	1.60 (1.20–2.30)	1.50 (1.05–2.27)

a *Prevalent cases defined as patients with a recorded diagnosis code for familial hypercholesterolaemia within the CPRD GOLD database.*

b *LLT defined as at least one prescription for either statin, ezetimibe, fibrates, PCSK9 inhibitors, niacin and bile acid sequestrants in the 6 months before the 30 June 2018. CPRD = Clinical Practice Research Datalink. IQR = interquartile range. LLT = lipid-lowering therapy. SD = standard deviation.*

The proportion of patients with recorded lipid measurements was higher for those with a prescription for a lipid-lowering therapy in the 6 months before the index date. For those with a lipid-lowering therapy prescription 2343 (88.5%) had a recorded measurement for total cholesterol versus 191 (65.5%) for those without, 1918 (72.5%) had a recorded measurement for LDL-C versus 710 (50.6%) without, and 260 (85.4%) had a recorded measurement for HDL-C versus 882 (62.9%). Mean levels were also lower for those treated with a lipid-lowering therapy in the 6 months before the index date compared with those untreated: 5.34 mmol/L (SD 1.50) versus 6.25 mmol/L (SD 1.55) for total cholesterol and 3.15 mmol/L (SD 1.34) versus 3.96 mmol/L (SD1.36) for LDL-C.

## Discussion

### Summary

This study describes the epidemiology, treatment, and lipid profiles of a population with familial hypercholesterolaemia and also estimated the prevalence of familial hypercholesterolaemia in the UK as 16.4 per 10 000 adult population, the equivalent of one patient per 608 population. This figure, based on a formal recorded diagnosis of familial hypercholesterolaemia using the Read code classification, is likely an underestimation, highlighting the need to better diagnose and identify patients in primary care in the UK. This study also reports that over one-third of patients diagnosed with familial hypercholesterolaemia were not treated, increasing their risk of experiencing cardiovascular events.

### Strengths and limitations

Although Read codes exist for statin intolerance, exploration indicated that these were used infrequently compared with the expected rates. In this study the authors therefore used a proxy definition of statin intolerance/contraindication based on prior statin exposure but without any statin prescriptions in the baseline period. However, some patients may not have had sufficient pre-baseline registration to identify prior statin exposure. Furthermore, patients may have been non-compliant rather than intolerant. As discussed below, this may be evidenced by the lower proportion of patients requiring secondary prevention (11.9%) classified as statin intolerance/contraindicated compared with patients requiring primary prevention (17.2%).

Data in CPRD is collected principally for the day-to-day administration of the primary care practice, thus data items are not recorded with the timeliness and precision that may be afforded a prospective register study. Identification of patients with familial hypercholesterolaemia was dependent on a medical code being recorded in the primary care dataset. Some diagnoses may have been recorded in text format or recorded with a less granular hypercholesterolaemia code and so the number of cases known to the primary care practice may have been underestimated. Notably, what criteria lay behind the recording of the diagnosis made by each practitioner is not known. It is possible that some may have used the electronic code provisionally to highlight potential patients with familial hypercholesterolaemia and thus the defined cohort may include some false positives.

### Comparison with existing literature

In this study the prevalence of familial hypercholesterolaemia was estimated as one per 608 population. This is approximately half the rate anticipated from other recent studies,^7^ which may themselves be conservative compared with prevalence studies based on prospective screening.^10^ This suggests that between 50% and 75% of patients with familial hypercholesterolaemia are undiagnosed, or at least not currently coded within the electronic health records of primary care systems. This proportion is similar to that reported by Iyen *et al*^24^ who used a combination of recorded diagnosis or clinical phenotype derived from the Simon Broome Register or the Dutch Lipid Clinic Network criteria to define a familial hypercholesterolaemia cohort. In this study, only 36.5% of the cohort were flagged by clinical diagnosis.

The efficacy of lipid-lowering therapy in familial hypercholesterolaemia has been established^25^^,^^26^ and yet for those patients with a diagnosis of familial hypercholesterolaemia over one-third had no evidence of lipid-lowering therapies prescribed within 6 months of the study index date. This is comparable with data from the Familial Hypercholesterolaemia Studies Collaboration (FHSC),^13^ which reported that 36.3% of patients with familial hypercholesterolaemia in the European population (excluding the Netherlands) were untreated with lipid-lowering therapy, whereas nearly half (47.4%) were untreated in the Netherlands cohort. Other studies have reported that nearly 50% of patients with familial hypercholesterolaemia are not prescribed statins.^10^^,^^27^ In contrast, the Royal College of Physicians familial hypercholesterolaemia register reported that 86% of adult patients with familial hypercholesterolaemia were prescribed statins.^15^

For patients in the secondary prevention group, this study showed the proportion of non-treated patients reduced to just over 10%. This perhaps unsurprising finding suggests that a prior history of a cardiovascular event is a bigger determinant of lipid-lowering therapy prescription than familial hypercholesterolaemia diagnosis. It is also noticeable that, with the definition of statin intolerance/contraindication used in the current study, there was a higher proportion of such patients in the primary prevention group when compared with the secondary prevention group. These observations may reflect the differing perceived risks of these patient groups, as patients without a prior cardiovascular event may be less risk-aware compared with patients who have experienced a cardiovascular event or diagnosis. Patients may also temporarily stop therapies if they achieve treatment targets and this decision may be informed by their perceived cardiovascular risk.

Patients untreated with a lipid-lowering therapy had higher median measurements of both LD-C (3.80 mmol/L versus 2.90 mmol/L) and total cholesterol (6.20 mmol/L versus 5.10 mmol/L) than those treated. However, lipids levels recorded in the current study appear to be lower than observed in other studies. In the FHSC,^13^ within the European population excluding the Netherlands, median LDL-C was 6.26 mmol/L versus 4.58 mmol/L and total cholesterol was 8.37 mmol/L versus 6.67 mmol/L for those untreated versus those treated. Interestingly, respective figures from patients in the Netherlands cohort in the FHSC study had values similar to the current study’s for total cholesterol (6.23 mmol/L versus 5.61 mmol/L) but higher for LDL-C (4.43 mmol/L versus 3.80 mmol/L). This may indicate that patients with recorded diagnoses of familial hypercholesterolaemia in the current study were skewed to patients with better LDL-C control although it may also suggest that some patients diagnosed with familial hypercholesterolaemia were false positives. As in the current study the authors only had access to prescription records derived from primary care, it is possible that some patients, particularly those with very high LDL-C or total cholesterol recordings, would be partly or solely managed in secondary care and initiated on lipid-lowering therapies in this setting, which would not be captured in CPRD. Local prescribing arrangements for PCSK9 inhibitors (alirocumab and evolocumab) recommend they should only be prescribed in secondary care and thus the recorded use of PCSK9 inhibitors in this study would likely be underestimated. It is not clear what proportion of patients this would apply to, but it was estimated in the US in 2019 that PCSK9 inhibitors were used in <1% of patients with either dyslipidaemia (including familial hypercholesterolaemia), elevated LDL-C (≥130 mg/dL [3.4 mmol/L]), or pre-existing CHD.^28^

Although the lipid levels reported in this study are below those reported elsewhere, both the treated and untreated subsets of the cohorts had median lipid levels above targets recommended by international guidelines.^11^ Interestingly, in the study by Iyen *et al*, mean lipid levels were higher, although this was in part self-fulfilling since approximately two-thirds of the cohort were selected on the basis of the Simon Broome Register or the Dutch Lipid Clinic Network criteria.^24^ Only a small proportion of patients were prescribed ezetimibe during the baseline period of this study despite ezetimibe being recommended where statins do not achieve effective lipid reduction.^29^ In this current cross-sectional study treatment pathways related to lipid levels are not reported and so it is not possible to determine whether ezetimibe was under-used.

### Implications for research and practice

As outlined in the NICE guidelines,^2^ it is important that patients with familial hypercholesterolaemia are identified within primary care as soon as possible. Electronic health records should be used to identify potential patients with familial hypercholesterolaemia who can have their diagnosis verified through genetic screening and then identify further cases through cascade screening.^1^ Accepting the caveats surrounding methods of recording and ascertainment, it appears that familial hypercholesterolaemia is under-recognised within primary care. The application of standard cardiovascular risk calculators for the general population is inappropriate as the lack of other risk factors, including age, which is an important driver of these algorithms, may not identify the true cardiovascular risk of this population. In this study median 10-year cardiovascular risk was estimated at <10% using the SCORE2 and ACC/AHA algorithms and 12.8% using the Framingham algorithms. If patients were not diagnosed with familial hypercholesterolaemia, a large proportion of these patients would be ineligible for lipid-lowering therapy based on cardiovascular risk criteria. This is a lost opportunity to initiate treatment for these patients and represents a long episode of undertreatment as follow-up testing would typically not take place for a further 5 years.

## Data

Due to the licence arrangements with CPRD, it is not possible to share data used in this study. This study is based in part on data from the CPRD obtained under licence from the UK Medicines and Healthcare products Regulatory Agency. The data are provided by patients and collected by the NHS as part of their care and support. The interpretation and conclusions contained in this study are those of the authors.
